# A high-fat jelly diet restores bioenergetic balance and extends lifespan in the presence of motor dysfunction and lumbar spinal cord motor neuron loss in TDP-43^A315T^ mutant C57BL6/J mice

**DOI:** 10.1242/dmm.024786

**Published:** 2016-09-01

**Authors:** Karen S. Coughlan, Luise Halang, Ina Woods, Jochen H. M. Prehn

**Affiliations:** Department of Physiology and Medical Physics, Centre for the Study of Neurological Disorders, Royal College of Surgeons in Ireland, 123 St. Stephen's Green, Dublin 2, Ireland

**Keywords:** Amyotrophic lateral sclerosis, High-fat jelly diet, Standard pellet diet, AMPK, Spinal cord, Motor neuron degeneration, TDP-43

## Abstract

Transgenic transactivation response DNA-binding protein 43 (TDP-43) mice expressing the A315T mutation under control of the murine prion promoter progressively develop motor function deficits and are considered a new model for the study of amyotrophic lateral sclerosis (ALS); however, premature sudden death resulting from intestinal obstruction halts disease phenotype progression in 100% of C57BL6/J congenic TDP-43^A315T^ mice. Similar to our recent results in SOD1^G93A^ mice, TDP-43^A315T^ mice fed a standard pellet diet showed increased 5′ adenosine monophosphate-activated protein kinase (AMPK) activation at postnatal day (P)80, indicating elevated energetic stress during disease progression. We therefore investigated the effects of a high-fat jelly diet on bioenergetic status and lifespan in TDP-43^A315T^ mice. In contrast to standard pellet-fed mice, mice fed high-fat jelly showed no difference in AMPK activation up to P120 and decreased phosphorylation of acetly-CoA carboxylase (ACC) at early-stage time points. Exposure to a high-fat jelly diet prevented sudden death and extended survival, allowing development of a motor neuron disease phenotype with significantly decreased body weight from P80 onward that was characterised by deficits in Rotarod abilities and stride length measurements. Development of this phenotype was associated with a significant motor neuron loss as assessed by Nissl staining in the lumbar spinal cord. Our work suggests that a high-fat jelly diet improves the pre-clinical utility of the TDP-43^A315T^ model by extending lifespan and allowing the motor neuron disease phenotype to progress, and indicates the potential benefit of this diet in TDP-43-associated ALS.

## Abstract (precis)

**Summary:** A high-fat diet extends lifespan in a TDP-43 mutant mouse model of motor neuron disease and might be a possible treatment strategy for individuals with amyotrophic lateral sclerosis.

## INTRODUCTION

Amyotrophic lateral sclerosis (ALS) is a severe neurodegenerative disease characterised by the selective weakening and loss of motor neurons eventually leading to paralysis and death. The prevalence of ALS worldwide is 2.6/100,000 and affected individuals have a median survival time of 22-48 months from diagnosis (Nelson, 1995; Chio et al., 2009; Logroscino et al., 2010). The majority of ALS cases are sporadic but 5-10% of cases are of genetic origin. Some of the identified causes of familial ALS are mutations in the genes encoding chromosome 9 open reading frame 72 (C9ORF72), fused in sarcoma (FUS), TAR DNA-binding protein 43 (TDP-43, also known as TARDBP) and super oxide dismutase 1 (SOD1) (DeJesus-Hernandez et al., 2011; Kwiatkowski et al., 2009; Vance et al., 2009; Arai et al., 2006; Rosen et al., 1993). Bioenergetic stress is a well-documented pathophysiological feature of ALS. Individuals with ALS are reported to have a lower body mass index (BMI), a hypermetabolic rate, mitochondrial clustering and hyperlipidaemia (Atsumi, 1981; Sasaki and Iwata, 1996; Siklos et al., 1996; Desport et al., 2005, 2001; Bouteloup et al., 2009; Dodge et al., 2013; Dupuis et al., 2008; Funalot et al., 2009; Kasarskis et al., 1996; Allen et al., 2015). Similarly, abnormal energy signalling is evident in ALS mouse models documented to have lower body mass across disease progression, accompanied by an increased metabolic rate, defective mitochondrial functioning, dysregulated oxidative phosphorylation and increased fatty acid uptake in muscles (Palamiuc et al., 2015; Coughlan et al., 2015; Kong and Xu, 1998; Wong et al., 1995; Bendotti et al., 2001; Jung et al., 2002; Mattiazzi et al., 2002; Kim et al., 2011).

5′ adenosine monophosphate-activated protein kinase (AMPK) is a master regulator and sensor of energy levels in the body and functions to promote energy homeostasis (Long and Zierath, 2006). When adenosine tri-phosphate (ATP) stores are low, AMPK becomes activated to restore energy levels. Recently, we detected elevated AMPK activation in lumbar spinal cords of SOD1^G93A^ mice from postnatal day (P)90 and P120 onwards, respectively (Coughlan et al., 2015). Furthermore, targeting AMPK pharmacologically using latrepirdine (an AMPK activator) resulted in extended lifespan and delayed onset of motor function deficits in SOD1^G93A^ mice (Coughlan et al., 2015).

Many pre-clinical ALS studies have been carried out in SOD1 mouse models; however, there is a great need to examine other genetic variants of ALS pathology. Recently, a novel TDP-43 mouse model of ALS expressing human transgene cDNA under the control of the murine prion promoter (mPrP) was created on a mixed C57BL/6;CBA genetic background (Borchelt and Sisodia, 1996; Wegorzewska et al., 2009). These mice were reported to live until ∼P154±19 and develop motor dysfunction at P120 (Wegorzewska et al., 2009). However, our group and other laboratories when examining these mice, now 100% congenic on a C57BL/6 background, observed premature sudden death (at ∼P100) prior to full development of neurodegenerative symptoms (Guo et al., 2012; Esmaeili et al., 2013; Medina et al., 2014; Hatzipetros et al., 2014). Autopsy results revealed this sudden death resulted from enteric dysfunction; specifically, pronounced dilation of the ileum and caecum linked to reduced nitric oxide synthase (NOS)-producing neurons in the terminal ileum and colon (Esmaeili et al., 2013; Herdewyn et al., 2014). Furthermore, vacuolisation of the myenteric plexus was reported along with a swollen intestinal wall (Guo et al., 2012; Esmaeili et al., 2013).

As energy imbalance is well documented in ALS and intestinal motility is compromised in this TDP-43 mouse model, we aimed to examine various dietary interventions to potentially alleviate bioenergetic stress and gastrointestinal (GI) difficulties. High-fat jelly diets have been shown to improve survival outcome in SOD1^G93A^ mice and individuals with ALS, whereas calorie restriction consistently worsens disease outcome (Pedersen and Mattson, 1999; Hamadeh et al., 2005; Dupuis et al., 2004; Wills et al., 2014). Therefore, we aimed to examine a standard pellet diet, low-fat jelly diet and a high-fat jelly diet, to decipher if any of these could ease defective bioenergetics and provide lifespan extension, allowing ALS neurodegenerative symptoms to develop in this model.

## RESULTS

### AMPK activity is increased in TDP-43^A315T^ mice sustained on a standard pellet diet

**Fig. 1.**
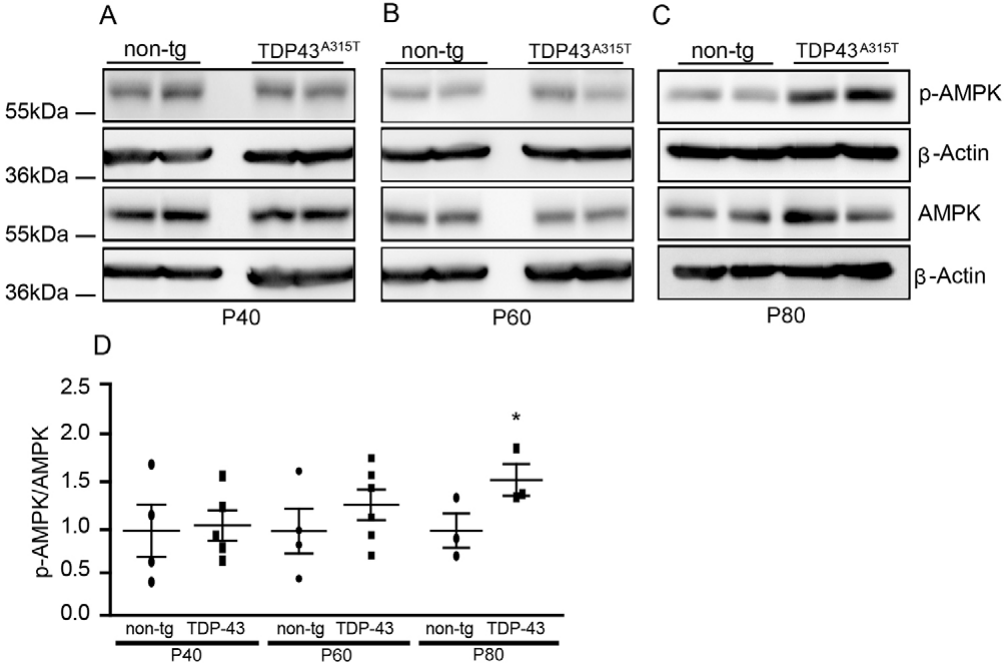
**AMPK activation is increased in P80 lumbar spinal cords of TDP-43^A315T^ mice sustained on standard pellet food.** (A-C) Representative western blots of total and phospho-AMPK protein levels in (A) P40 non-tg (*n*=4) and tg (*n*=5), (B) P60 non-tg (*n*=4) and tg (*n*=6), and (C) P80 non-tg (*n*=3) and tg (*n*=3) lumbar spinal cords. (D) Densitometry quantification of phospho-AMPK/AMPK relative to β-actin, where a significant increase in the phospho-AMPK to total AMPK ratio was observed at P80 (*P=0.0298, two-tailed *t*-test). Data are presented as mean±s.e.m. and statistically analysed by independent samples Student's *t*-tests. *n* numbers represent biological replicates (each a separate spinal cord from individual animals). Each respective value was normalised to the relevant β-actin loading control. Please note, some of the β-actin blots in Fig. 1A,B are also shown in Fig. 4A,B owing to triple probing of membranes.

AMPK activation generates energy sources when necessary and its activity level reflects energy status in tissues (Long and Zierath, 2006). We previously measured AMPK levels in SOD1^G93A^ mice throughout disease progression and observed increased AMPK activation from disease onset (P90) onwards (Coughlan et al., 2015). Therefore, we were interested in investigating AMPK levels in TDP-43^A315T^ mice. As TDP-43^A315T^ mice on a 100% congenic C57BL6/J background given a standard pellet diet live until ∼P100, we examined P40, P60 and P80 time points. Lumbar spinal cord homogenates from transgenic (tg) and non-tg specimens at each time point were collected and probed for threonine-172 phosphorylated and total AMPK protein levels via western blotting. We found significantly increased AMPK activity at P80, whereas no significant change in AMPK activation was noted at the earlier time points P40 and P60 (Fig. 1).

### A high-fat jelly diet extended lifespan in TDP-43^A315T^ mice in the presence of a motor neuron disease phenotype

In an effort to alleviate the bioenergetic stress in the TDP-43^A315T^ mouse model identified through AMPK profiling (Fig. 1) and suggested by pharmacological targeting with latrepirdine (Fig. S1), we next trialled various diet regimes and examined survival rates in male mice (Coughlan et al., 2015; Esmaeili et al., 2013; Guo et al., 2012; Hatzipetros et al., 2014). In one approach, we fed mice with a low-fat jelly diet (73% moisture) for ease of digestion and absorption to alleviate GI dysfunction that might be the cause of the bioenergetic stress in this model (Esmaeili et al., 2013; Guo et al., 2012; Hatzipetros et al., 2014). In a second approach, we trialled a high-fat jelly diet (30% moisture) in an effort to alleviate GI dysfunction but additionally to address the bioenergetic stress previously identified (Fig. 1). The caloric content of the three diets used is shown in Table S1.

**Fig. 2.**
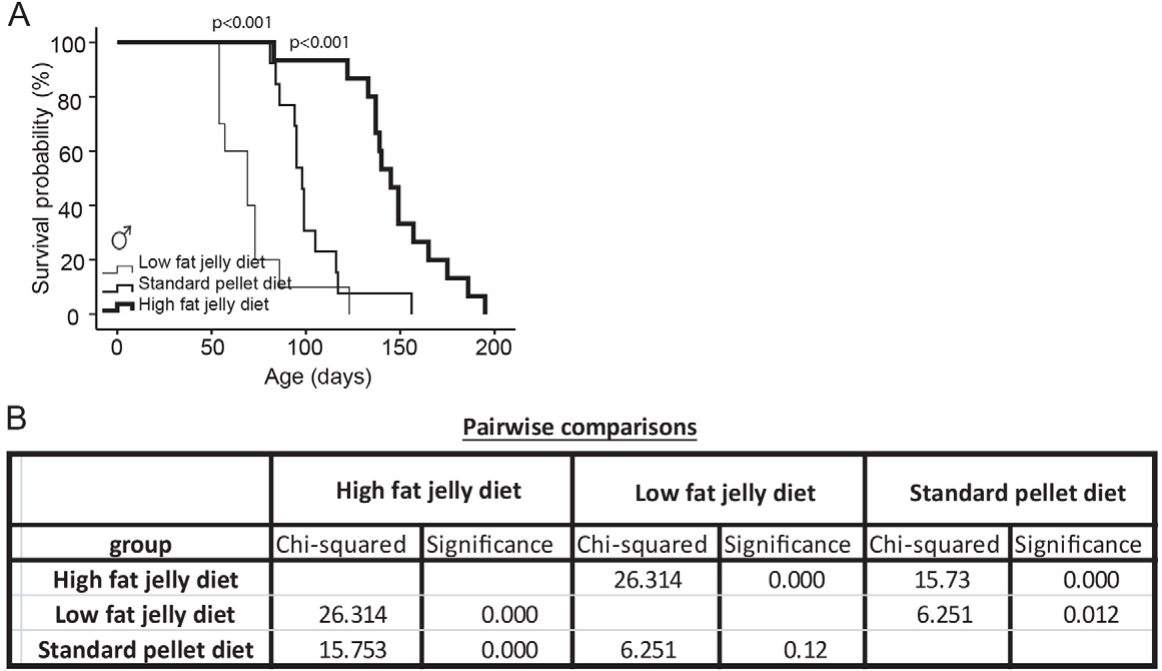
**A high-fat jelly diet extends lifespan in TDP-43^A315T^ mice.** Of note, only the lifespan of male mice was examined. (A) Kaplan–Meier analysis of probability of survival of standard pellet-fed tg mice (*n*=14) compared with low-fat jelly diet-fed tg mice (*n*=10) and high-fat jelly diet-fed tg mice (*n*=15). A high-fat jelly diet significantly extended survival in tg mice compared with standard pellet-fed tg mice where high-fat jelly fed mice lived for an average of 147±27 days (mean±s.e.m.) and standard pellet-fed mice lived for 102±19 days. A low-fat jelly diet significantly decreased lifespan in tg mice compared with standard pellet-fed mice where low-fat jelly fed mice lived for an average of 71±21 days. (B) Pairwise comparisons [log rank (Mantel–Cox)] showing chi-squared and significance values for high-fat jelly diet versus low-fat jelly diet (chi-squared=26.314 and significance *P*=1.375948×10^−6^), high-fat jelly diet versus standard pellet (chi-squared=15.73 and significance *P*=7.217339×10^−5^) and low-fat jelly diet versus standard pellet (chi-squared=6.251 and significance *P*=0.012).

TDP-43^A315T^ mice fed a high-fat jelly diet showed considerable survival extension with mice living to ∼147±27 days (mean±s.d.) in comparison with standard pellet-fed mice living for an average of 102±19 days (*P*<0.001; Fig. 2). In contrast, the low-fat jelly diet did not result in survival extension, in fact on this diet there was a reduced lifespan of 71±21 days (*P*<0.001; Fig. 2).

### Delayed AMPK activation in TDP-43^A315T^ mice sustained on a high-fat jelly diet

**Fig. 3.**
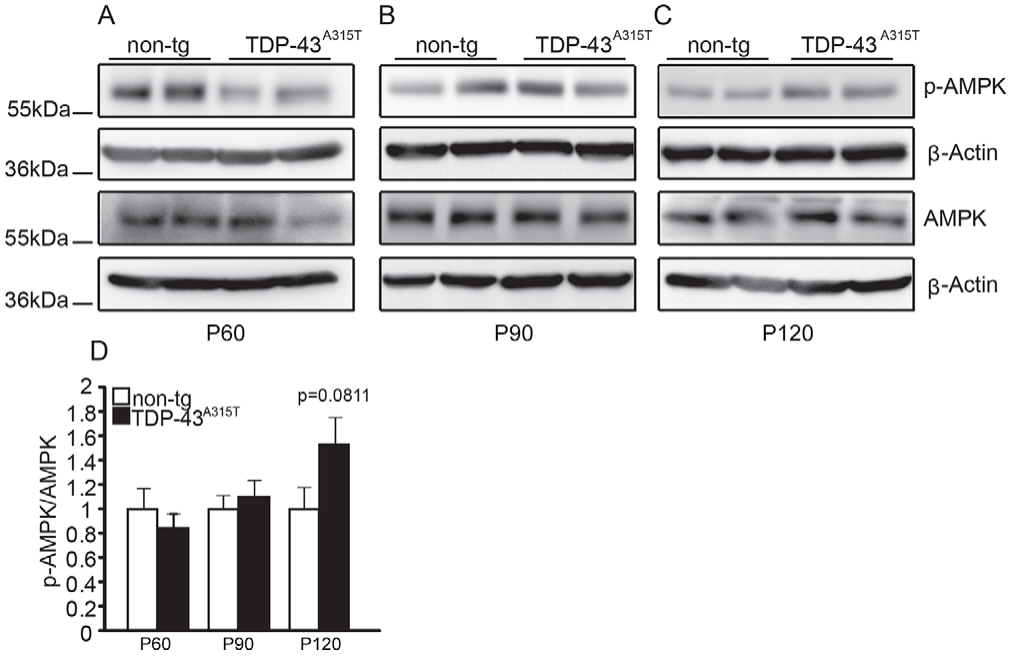
**High-fat jelly diet delays activation of AMPK in lumbar spinal cords of TDP-43^A315T^ mice compared** **with** **non-tg controls.** (A-C) Representative western blots of total and phospho-AMPK protein levels in (A) P60 non-tg (*n*=4) and tg (*n*=5) (three replicates of the experiment combined), (B) P90 non-tg (*n*=6) and tg (*n*=8) (three replicates of the experiment combined), and (C) P120 non-tg (*n*=5) and tg (*n*=4) (two replicates of the experiment combined). (D) Quantification of phospho-AMPK/AMPK levels relative to β-actin across disease progression. No significant change was observed across disease progression in phospho-AMPK, total AMPK or phospho-AMPK/AMPK protein levels. Data are presented as mean±s.e.m. and statistically analysed by two-tailed Student's *t*-tests. *n* numbers represent biological replicates (each a separate spinal cord from individual animals). Tg values are displayed relative to non-tg controls and values normalised to β-actin loading controls. Please note, the same β-actin blot in Fig. 3A is also shown in Fig. 4D owing to triple probing of membranes.

Similar to the AMPK activation investigation in Fig. 1, we harvested lumbar spinal cords from non-tg and tg TDP-43^A315T^ mice; however, these mice were fed a high-fat jelly diet from weaning (∼P30). As these mice lived for an average of 147 days on a high-fat jelly diet we examined P60, P90 and P120 time points and probed for active, threonine-172 phosphorylated and total AMPK protein levels via western blotting. We found no significant difference in AMPK activation at any time point examined; however, there was a trend towards elevated AMPK activity at the late time point P120 (*P*=0.0811; Fig. 3). Comparing these results with the results in Fig. 1 suggests that a high-fat jelly diet delays AMPK activation in TDP-43^A315T^ mice.

Decreased ACC phosphorylation early in disease progression in high-fat jelly diet fed TDP-43A315T mice compared with standard pellet-fed mice.

**Fig. 4.**
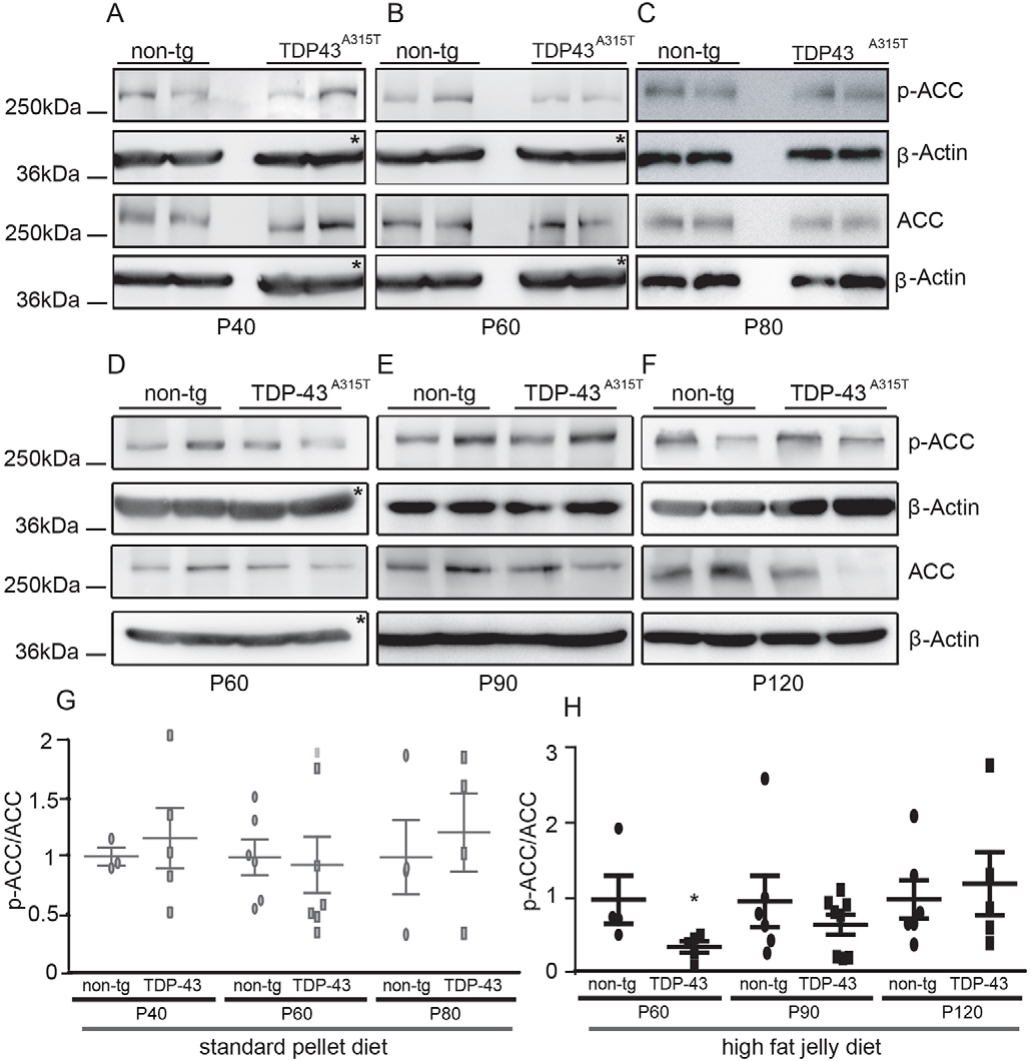
**Decreased fatty acid oxidation early in disease progression in high-fat jelly fed TDP-43^A315T^ mice compared** **with** **standard pellet-fed mice.** (A-F) Mice were weaned to their particular diet at ∼P30. Representative western blots of total and phospho-ACC protein levels in (A) P40 non-tg (*n*=3) and tg (*n*=5), (B) P60 non-tg (*n*=6) and tg (*n*=7), (C) P80 non-tg (*n*=3) and tg (*n*=4) lumbar spinal cords of standard pellet-fed mice; and (D) P60 non-tg (*n*=4) and tg (*n*=5), (E) P90 non-tg (*n*=6) and tg (*n*=8), (F) P120 non-tg (*n*=5) and tg (*n*=5) lumbar spinal cords of high-fat jelly diet mice. (G,H) Quantification of phospho-ACC/ACC levels relative to β-actin across disease progression of standard pellet- (G) and high-fat jelly diet-fed mice (H). P40 (standard pellet diet) versus P60 (high-fat jelly diet) showed statistically different levels of ACC phosphorylation (Student's *t*-test, *P=0.0338). Additionally, P60 (high-fat jelly diet) was comparatively different to P120 (high-fat jelly diet) (one-way ANOVA, *P*=0.0305; non-tg P60 versus tg P60, *P*=0.070, two-tailed). Data are presented as mean±s.e.m. and statistically analysed by Student's *t*-tests. Tg values are displayed relative to non-tg controls and values are normalised to β-actin loading control. Please note, in Fig. 4A,B the same β-actin is shown as in Fig. 1A,B and similarly, in Fig. 4D the same β-actin is shown in Fig. 3A owing to triple probing of membranes (asterisks).

ACC phosphorylation, via AMPK activation, leads to increased fatty acid oxidation and increased ATP production. This process of lipid breakdown is elevated when glucose levels are low (Foster, 2012). Examination of ACC phosphorylation showed decreased levels of ACC activity at P60 when mice were maintained on a high-fat jelly diet in comparison to P40 standard pellet-fed mice (*P*=0.0338, Fig. 4). This implies a diminished need for energy production and decreased fatty acid oxidation at early stage time points in TDP-43 disease progression while on a high-fat supplemented diet. Moreover, one-way ANOVA of all time points (P60, P90 and P120) of TDP-43 mice fed a high-fat jelly diet showed a significant elevation in ACC phosphorylation rate at P120 versus P60 indicating an increased need for energy production in late stage disease progression.

### A high-fat jelly diet does not rescue the enteric phenotype in the TDP-43^A315T^ mouse model

**Fig. 5.**
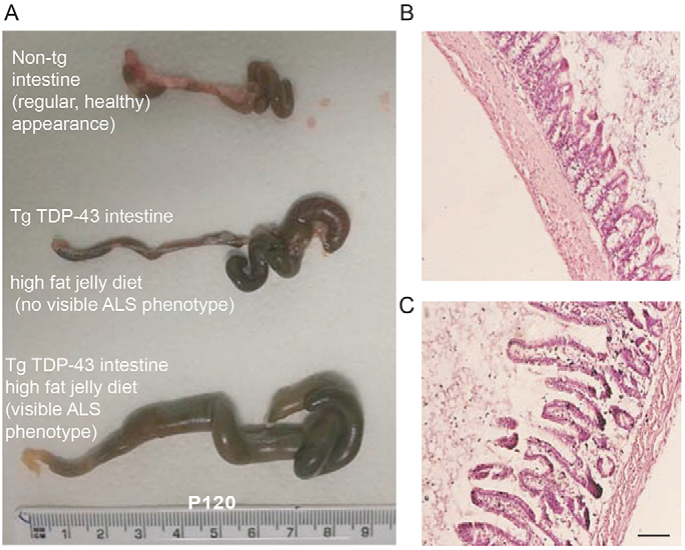
**Intestinal staining in non-tg and TDP-43^A315T^ tg mice sustained on a high-fat jelly diet.** (A) Images representing intestinal morphology at P120 (late disease stage), in a high-fat jelly diet non-tg mouse, a TDP-43^A315T^ tg high-fat jelly diet mouse that did not show a disease phenotype at P120, and a litter-matched TDP-43^A315T^ tg high-fat jelly diet mouse that did show a severe ALS disease phenotype at P120. The TDP-43^A315T^ tg mouse that was not sick still showed intestinal enlargement. The sick TDP-43^A315T^ tg mouse had a grossly distended gut. (B) H&E staining in the small intestine of a non-tg mouse and a TDP-43^A315T^ tg high-fat jelly fed mouse at end stage (approximately P150). Note that the TDP-43^A315T^ tg small intestine was swollen. Scale bar: 100 µm.

Our research group has unpublished data, supported by published work from other research groups, showing reduced colonic propulsions, enlarged caecum and swollen intestines in tg TDP-43^A315T^ mice on a standard pellet diet (Esmaeili et al., 2013; Herdewyn et al., 2014). We therefore examined the intestinal phenotypes in high-fat jelly diet-fed TDP-43^A315T^ mice, and noted various degrees of distended intestines in tg mice compared with non-tg mice. Fig. 5A displays intestinal structures from a non-tg mouse on a standard pellet diet, a tg intestine on a high-fat jelly diet not showing any ALS phenotype and a tg intestine on a high-fat jelly diet showing a severe ALS phenotype. All of these mice were males, littermates and sacrificed at P120, demonstrating the variability in the model even with the high-fat jelly diet. Representative images from H&E staining of the small intestine demonstrate the swollen small intestine of a tg mouse on the high-fat jelly diet compared with the healthy control (Fig. 5B,C).

**Fig. 6.**
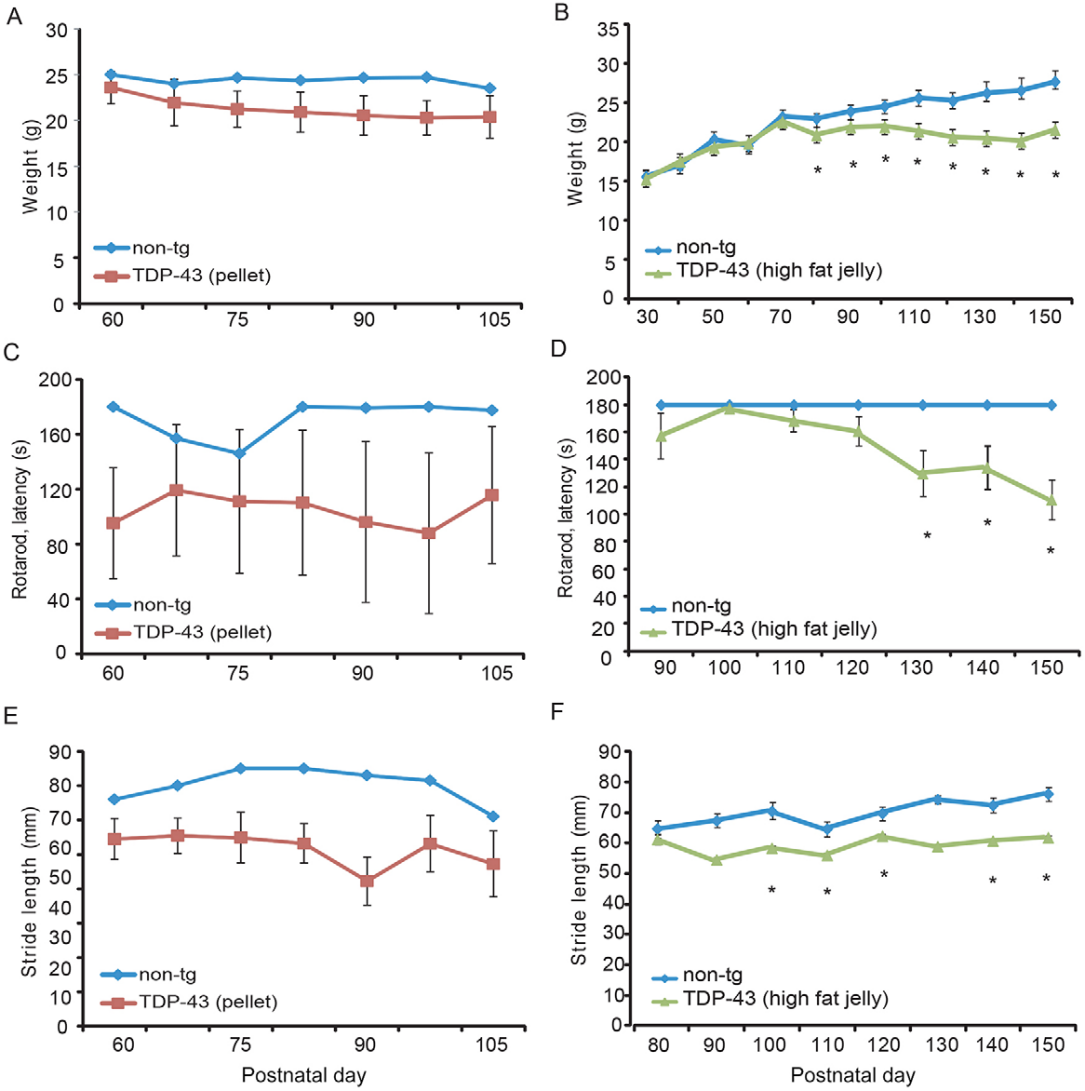
**ALS disease phenotype develops and progresses in later stages of TDP-43^A315T^ disease course when fed** **a high-fat jelly** **diet.** (A,B) Non-tg and TDP-43^A315T^ tg weight measurements recorded across disease progression in mice fed a standard pellet diet (A) and high-fat jelly diet (B). No change in weight was observed in non-tg versus tg mice over time in standard pellet-fed mice, but high-fat jelly diet-fed TDP-43^A315T^ tg mice weighed significantly less than their non-tg counterparts from P80 onwards (*P<0.05). (C,D) Non-tg and TDP-43^A315T^ tg Rotarod recordings across disease progression in mice fed a standard pellet diet (C) and high-fat jelly diet (D). Motor function deficits were significantly evident in high-fat jelly diet-fed TDP-43^A315T^ tg mice at P130 (*P=0.003), P140 (*P=0.017) and P150 (*P=0.006). The data did not show a significant decrease in motor function abilities at P160 onwards; however, this was likely owing to the small population of mice still alive skewing the results at these later time points (data not included on graph). (E,F) Non-tg and TDP-43^A315T^ tg stride length analysis in mice fed a standard pellet diet (E) and high-fat jelly diet (F). Stride length was significantly decreased in high-fat jelly diet-fed TDP-43^A315T^ tg mice from P100 (*P<0.05) onwards compared with non-tg littermates. Standard pellet diet, non-tg *n*=2 and TDP-43^A315T^ tg *n*=11; high-fat jelly diet, non-tg and TDP-43^A315T^ tg *n*≥5 at each time point shown, but *n*=3 at P90). Data are presented as mean±s.e.m. and statistically analysed by unpaired Student *t*-tests where possible.

Progressive motor dysfunction owing to lifespan extension is evident in TDP-43^A315T^ mice fed a high-fat jelly diet compared with a standard pellet diet (Fig. 6C-F). The lengthened lifespan of the TDP-43^A315T^ mice maintained on high-fat jelly diet allowed us to measure motor function performance throughout disease progression in these mice. We also included motor function data from non-tg mice sustained on a standard pellet diet; however, there were only two mice in these groups, and therefore, statistical tests were not carried out on these data. These graphs provide visual comparisons between the two diet groups. Weight analysis revealed a significant weight loss in tg animals versus non-tg mice at P80 and onwards (Fig. 6B) when sustained on a high-fat jelly diet, whereas no change in weight seems to occur when non-tg mice on standard pellet diet are compared with tg counterparts. Furthermore, Rotarod measurements showed no change over time in tg mice sustained on a standard pellet diet, whereas significantly decreased abilities in tg mice on a high-fat jelly diet were noted at P130 (*P*=0.003), P140 (*P*=0.017), P150 (*P*=0.006) (Fig. 6D) compared with non-tg healthy controls. Gait analysis measurements evidenced weakened stride length abilities from P100 onwards in the high-fat jelly fed tg mice (Fig. 6F) that was similar in tg mice on a standard pellet diet (Fig. 6E).

### Motor dysfunction linked to decreased lumbar motor neuron survival in TDP-43^A315T^ mice at P120 (on a high-fat jelly diet)

**Fig. 7.**
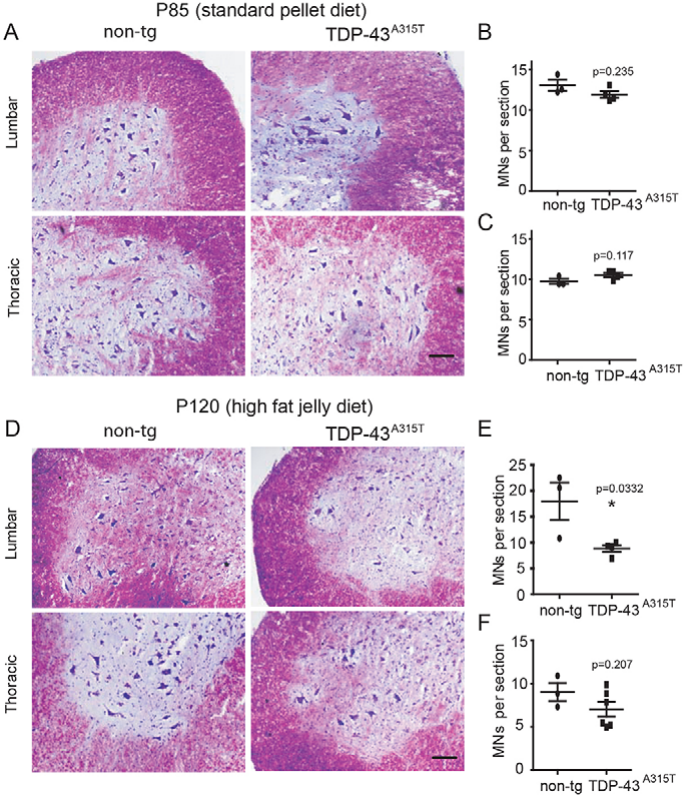
**TDP-43^A315T^ mice (high-fat jelly diet) show significant loss in motor neuron survival at P120 in the lumbar spinal cord region.** (A) Representative images of ∼P85 non-tg (*n*=3) and TDP-43^A315T^ tg (*n*=4) thoracic and lumbar spinal cords (standard pellet diet). (B) P85 lumbar spinal cord motor neuron counts where no difference in motor neuron survival was observed (*P*=0.235, two-tailed, equal variance assumed). (C) P85 thoracic spinal cord motor neuron counts, no difference in motor neuron survival was observed (*P*=0.117, two-tailed). (D) Representative images of P120 non-tg (*n*=3) and TDP-43^A315T^ tg (*n*=4-6) thoracic and lumbar spinal cords. (E) P120 lumbar spinal cord motor neuron counts where a significant decrease in motor neuron survival was observed (*P*=0.0332, two-tailed). (F) P120 thoracic spinal cord motor neuron counts, no significant difference observed (*P*=0.207, two-tailed, equal variance assumed). End-stage spinal cords were also analysed; however, no significant difference in motor neuron survival was detected at this time point (data not shown). Data is represented as mean±s.e.m. and analysed by unpaired Student's *t*-test. Scale bar: 100 µm.

Analysis of motor neuron survival was carried out via Nissl staining in lumbar (L1-L5, 20 µm) and thoracic (T5-T10, 20 µm) spinal cord sections at P85 in standard pellet-fed mice and P120 (late stage disease progression) and P150 (approximate end stage) time points in high-fat jelly fed non-tg and TDP-43^A315T^ mouse specimens. Motor neurons were identified as large (width >30 µm), multi-polar structures with dark blue or purple staining. No difference in motor neuron survival was noted between non-tg and tg mice fed a standard pellet diet in lumbar or thoracic sections (P85) (Fig. 7A-C). We observed a significant decrease in motor neuron survival at P120 in the lumbar spinal cords region sustained on a high-fat diet (Fig. 7D,E), whereas no change in motor neuron counts were detected in standard pellet-fed mice (lumbar or thoracic sections), thoracic P120 samples (Fig. 7D,F) or lumbar or thoracic P150 samples (data not shown).

## DISCUSSION

Our results show that targeting bioenergetic stress in TDP-43^A315T^ mice via a high-fat jelly diet alleviated high energetic demands as evidenced by delayed AMPK activation and decreased ACC phosphorylation. In turn, the high-fat jelly diet regime had a pro-survival effect in TDP-43^A315T^ mice with lifespan extension to 147 days on average. This prolonged survival rate allowed a progressive motor neuron disease phenotype to develop as evidenced by weight loss at P80 and onwards, diminished Rotarod abilities observed at P130, 140 and 150 and decreased gait abilities recorded at P100 and onwards. The weakness and loss of limb function identified was associated with lumbar motor neuron loss. The high-fat jelly diet regimen did not rescue the previously documented enteric dysfunction in this model, however. A high-fat jelly diet therefore significantly improves the pre-clinical utility of the TDP-43^A315T^ model, and suggests a beneficial role for high-fat jelly diet in TDP-43-associated ALS.

This work was carried out to examine energy status and nutrient utilisation in TDP-43^A315T^ mice in the same way as we previously documented bioenergetic imbalance in SOD1^G93A^ mice (Coughlan et al., 2015). We found that targeting TDP-43^A315T^ energetic imbalance with a high-fat jelly diet delayed AMPK activation and as a result, decreased the need for energy production (evidenced by reduced ACC fatty acid oxidation), which overall led to a prolonged lifespan. Delayed AMPK activation and a pro-survival effect with a high-fat jelly diet has previously also been observed in SOD1^G93A^ and SOD1^G68R^ mice (Zhao et al., 2015; Dupuis et al., 2004). Additionally, we demonstrated that pharmacologically targeting energy stress signalling with the AMPK activator latrepirdine improved survival outcome in TDP-43^A315T^ mice. Hyperlipidaemia, hypermetabolic rate and metabolic dysfunction are well-characterised pathophysiological features of early ALS disease progression that could explain the bioenergetic stress and AMPK activation we observed in these mice (Dupuis et al., 2004; Desport et al., 2001; Funalot et al., 2009; Bouteloup et al., 2009). Moreover, most nutrient absorption takes place in the small intestine; however, TDP-43^A315T^ mice characteristically experience severe enteric dysfunction that could lead to depleted nutrient availability later in disease course.

AMPK activation leads to ACC phosphorylation. We observed a significant increase in AMPK activation at P80 in pellet-fed mice; however, this increase was not mirrored in our ACC P80 data. However, previous studies from our laboratory have revealed similar patterns to this where AMPK activation was followed by delayed ACC activity at a later time point (Coughlan et al., 2015). In the high-fat jelly fed mice the AMPK and ACC phosphorylation patterns were similar. It is possible that levels of phosphorylated or non-phosphorylated ACC are subject to other regulatory processes during disease progression.

Although we could not measure exact caloric intake, mice who were subjected to a high-fat jelly diet lived the longest, whereas a low-fat jelly diet alone actually worsened disease condition (71 days approximate lifespan), indicating that a high-calorie and high-fat diet was the key element necessary to relieve bioenergetic stress and to incur a pro-survival effect in TDP-43^A315T^ mice. The high-fat jelly diet did alleviate disease state as generally transgenic mice did not die suddenly and lived longer; however, at end stage most transgenic mice had markedly swollen intestinal structures. The TDP-43^A315T^ mouse model on a high-fat jelly diet is an easily adaptable model with a progressive ALS phenotype and identifiable lumbar motor neuron loss. There is an increasing need for, but limited availability of, ALS mouse models. Additionally, there is a need to move away from mutant SOD1 mouse models alone to account for disease heterogeneity and allow treatment validation in more than one model (Ludolph et al., 2010). We here demonstrate that with an easy nutritional intervention, the TDP-43^A315T^ mouse model, which is widely distributed in the community, can be easily adapted for future pre-clinical trials.

There is a growing body of research on nutritional status and dietary interventions for the treatment of ALS, with a focus on high-fat and hypercaloric supplements providing benefits in patient wellbeing and survival (Al-Chalabi, 2014). A recent study with ∼1 million participants, of which 995 had ALS, showed a 34% reduced risk of developing ALS associated with dietary *n*-3 polyunsaturated fatty acid (PUFA) intake (Fitzgerald et al., 2014). Similarly, a study of 132 individuals with ALS and 220 healthy controls demonstrated a 60% decreased risk in ALS development with PUFA intake (>32 g PUFA group versus the lowest group <25 g PUFA; Veldink et al., 2007). Dorst and colleagues reported a significant increase in lifespan in individuals with ALS with higher triglyceride levels (Dorst et al., 2011). A low-fat, high-carbohydrate diet has been associated with a higher risk of developing ALS, whereas high fat consumption has been recorded as a factor that reduces the risk of developing ALS (Okamoto et al., 2007). Furthermore, a randomised, double-blinded placebo-controlled phase 2 trial of 24 individuals with ALS in late stage disease course showed improved survival on a high-calorie diet (Wills et al., 2014). Interestingly, lipid peroxidation, the degradation of lipids, has been reported to be increased in sporadic individuals with ALS, correlating with extent of disease (Simpson et al., 2004). Evidence also suggests that lipid-lowering drugs (e.g. statins) correlate with disease risk and should be given with considered caution in ALS patients (Dorst et al., 2011; Nefussy et al., 2011). Our preclinical study in TDP-43^A315T^ mice demonstrates that bioenergetic stress and AMPK signalling is attenuated through a high-fat diet at the level of the diseased tissue, and suggests that nutritional intervention using a high-fat and high-calorie diet might represent a therapeutic avenue in individuals with TDP-43 ALS.

## MATERIALS AND METHODS

### Animals

Prp-hTDP-43 (A315T) transgenic mice were purchased from Jackson Laboratory (Bar Harbor, ME, USA). Of note, Jackson Laboratory reports that the background of these mice is now fully congenic on a C57BL6/J background. Mice were housed in cages containing between three and five mice and kept at constant temperature (22°C) on a 12 h light/dark cycle (07:00 h on, 19:00 h off), with *ad libitum* food and water available. Only male mice were used in this study to avoid sex variation. All experiments were carried out under license (no. B100/4414) from the Department of Health and Children, Ireland, with ethical approval from the Royal College of Surgeons in Ireland Research Ethics Committee (REC625b).

### Treatment with AMPK activator latrepirdine

Latrepirdine (Medivation, San Fracisco) stock was dissolved in 1× PBS (vehicle). Non-tg and tg TDP-43^A315T^ mice (sustained on a standard pellet diet) were administered latrepirdine (1 μg/kg/d, intraperitoneal injection) or vehicle from P40 until P80, and survival was assessed. Animals were randomly assigned to either vehicle or latrepirdine groups.

### Animal diets

Three sets of diets were trialled in this study; a standard rodent pellet diet (2018 X Tekland Global diet, Harlan Laboratories, Houston, TX, USA), a low-fat jelly diet (DietGel 76A, ClearH20, Westbrook, ME, USA) and high-fat jelly diet (DietGel Boost, ClearH20). Mice were weaned at P30 to P35 and placed on the respective diet for their study group. The standard pellet diet contained 24% protein, 18% fat and 58% carbohydrate but contains no moisture. The low-fat jelly diet is composed of 4.7% protein, 1.5% fat, 18% carbohydrate and 73% moisture. The high-fat jelly diet nutritional breakdown is 9.9% protein, 21.6% fat, 37.8% carbohydrate and 30% moisture. Table S1 displays the macronutrient breakdown of the three diets and caloric content of each.

### Assessment of lifespan and disease progression *in vivo*

For lifespan and motor function assessment, animals were age-, sex- and litter-matched in accordance with recent ALS guidelines for the generation of preclinical data (Ludolph et al., 2010). Mice were trained for use of motor function equipment before testing measurements commenced. Motor function tests included Rotarod (Stoelting, Wood Dale, IL, USA), and stride length recordings (Gurney et al., 1994). Weight measurements and Rotarod tests were performed bi-weekly, whereas stride length measurements were recorded once a week. All tests were carried out by the same blinded observer and interval rest days were employed in between motor function testing days*.* End stage of ALS disease progression was determined by a number of factors: a ‘swimming’ or ‘waddling’ gait (documented in Movie 1), a hunched posture, piloerection, lack of grooming, anti-social behaviour, noticeable weight loss, and intestinal enlargement and/or discomfort.

### Assessment of motor neuron survival *in vivo*

Cryoprotected thoracic and lumbar spinal cord samples were sectioned (20 µm) on the cryostat from T5-T10 and L1-L5 and Nissl stained with Cresyl Violet (0.1%). Nissl-positive motor neuron cells were counted (according to the pre-determined inclusion criteria – cell bodies must be between 30 and 80 μm in diameter, have a dark nucleolus and be multi-polar in structure) in every third section of the ventral horn region of spinal cords and motor neuron survival assessed.

### Assessment of intestinal histopathology

Fixed-paraffin embedded intestinal tissue was sectioned (10 µm) using the microtome with a specific focus on the ileocaecal junction and small intestine regions owing to identified pathological changes in these areas of the gastro-intestinal tract in previous publications (Herdewyn et al., 2014; Esmaeili et al., 2013; Guo et al., 2012). Intestinal tissue was stained with Haematoxylin and Eosin (H&E) dyes to visualise relevant structures.

### Western blotting

Samples were homogenised in RIPA buffer (50 nM Tris-HCl pH 7.4, 1% NP-40, 0.25% Na-deoxycholate, 150 mM NaCl, 1 mM EDTA) supplemented with protease inhibitor mixture (1:100; Sigma) and phosphatase inhibitors (1:100; Sigma). Equal amounts of protein were diluted in Laemmli buffer, separated by SDS-PAGE and transferred to a nitrocellulose membrane using semi-dry transfer apparatus. Membranes were blocked using 4% bovine serum albumin (BSA) in TBS-Tween 20 (0.1%; Sigma) for 1 h at room temperature. Membranes were then incubated with the following antibodies: anti-threonine-172-phospho-AMPK (1:1000; 2535s, Cell Signaling Technology), anti-AMPK (1:1000; 2532s, Cell Signaling Technology), anti-phospho-ACC (1:1000; 3661s, Cell Signaling Technology), anti-ACC (1:1000; 3676s, Cell Signaling Technology), anti-β-actin (1:5000; A3853, Sigma), made up in 1% BSA, overnight at 4°C. After washing, the membranes were incubated with horseradish peroxidase (HRP)-conjugated secondary antibodies [1:5000; 111-035-144 (anti-rabbit IgG) or 115-035-062 (anti-mouse IgG), Jackson ImmunoResearch] for 1 h at room temperature. Membranes were washed and incubated with enhanced chemiluminescence substrate (Millipore) and imaged using the LAS 4000 Reader (Fujifilm). Densitometric analysis was performed in ImageJ software (NIH) on all samples and values were normalised to loading control (β-actin) and standardised to the corresponding non-tg control.

### Statistical analysis

All data are presented as mean±s.e.m. Determining whether data was parametric or non-parametric was subject to the power (i.e. depending on the sample number) and the distribution of the data. Therefore, if sample numbers were greater than five per treatment or genotype group a histogram representation of the data was used to determine whether the data was normally distributed (bell-shaped curve) or abnormally distributed. As low sample numbers (less than five per group) were too small to test for normality, the standard tests assuming normal distribution were employed. For normally distributed data i.e. parametric data, when only two groups were analysed, a two-tailed Student *t*-test was used (equal variances assumed). Otherwise, the two-tailed Mann–Whitney U-test was used.

Survival results were assessed by the Kaplan–Meier estimate test. The Kaplan–Meier test assumes normal distribution and is one of the best methods to analyse the fraction of subjects in a group living post treatment (Goel et al., 2010). As the Kaplan–Meier estimate assumes normal distribution we consistently examined our pre-clinical study data using tests that assume normal distribution. Motor function tests were analysed by one-way ANOVA and Tukey's post hoc test.

Statistical analysis was performed by PASW statistics version 22 Software (IBM, Dublin, Ireland) or GraphPad Prism (GraphPad Software, Inc.) and significance was incurred at *P*<0.05.
